# Prevalence of Children with Severe Fetal Alcohol Spectrum Disorders in Communities Near Rome, Italy: New Estimated Rates Are Higher than Previous Estimates

**DOI:** 10.3390/ijerph8062331

**Published:** 2011-06-22

**Authors:** Philip A. May, Daniela Fiorentino, Giovanna Coriale, Wendy O. Kalberg, H. Eugene Hoyme, Alfredo S. Aragón, David Buckley, Chandra Stellavato, J. Phillip Gossage, Luther K. Robinson, Kenneth Lyons Jones, Melanie Manning, Mauro Ceccanti

**Affiliations:** 1 Center on Alcoholism, Substance Abuse, and Addictions (CASAA), The University of New Mexico, 2650 Yale SE, Albuquerque, NM 87106, USA; E-Mails: wkalberg@unm.edu (W.O.K.); asaragon@salud.unm.edu (A.S.A.); dbuckley@unm.edu (D.B.); cbs0920@unm.edu (C.S.); jgossage@unm.edu (J.P.G.); 2 The University of Rome, “La Sapienza”, Rome 00186, Italy; E-Mails: d.fiorentino@libero.it (D.F.); gcoriale@tin.it (G.C.); Mauro.Ceccanti@uniroma1.it (M.C.); 3 Sanford School of Medicine, University of South Dakota, Sioux Falls, SD 57104, USA; E-Mail: Gene.Hoyme@sanfordhealth.org; 4 School of Medicine, State University of New York at Buffalo, Buffalo, NY 10138, USA; E-Mail: Dysmorph@aol.com; 5 School of Medicine, University of California at San Diego, San Diego, CA 94109, USA; E-Mail: klyons@ucsd.edu; 6 Stanford University School of Medicine, Palo Alto, CA 94109, USA; E-Mail: mmanning@stanford.edu

**Keywords:** fetal alcohol spectrum disorders (FASD), fetal alcohol syndrome (FAS), epidemiology, prevalence, Italy, alcohol consumption

## Abstract

**Objective:**

To determine the population-based epidemiology of fetal alcohol syndrome (FAS) and other fetal alcohol spectrum disorders (FASD) in towns representative of the general population of central Italy.

**Methods:**

Slightly revised U.S. Institute of Medicine diagnostic methods were used among children in randomly-selected schools near Rome. Consented first grade children (n = 976) were screened in Tier I for height, weight, or head circumference and all children ≤10th centile on one of these measurements were included in the study. Also, teachers referred children for learning or behavioral problems. Children meeting either of these two criteria, along with randomly-selected controls, advanced to Tier II which began with a dysmorphology examination. Children with a possible FASD, and controls, advanced to Tier III for neurobehavioral testing, and their mothers were interviewed for maternal risks. Final diagnoses using indicators of dysmorphology, neurobehavior, and maternal risk were made in formally-structured, interdisciplinary case conferences.

**Results:**

Case control comparisons of physical, neurobehavioral, and maternal risk variables are presented for 46 children with an FASD and 116 randomly-selected controls without a diagnosis on the FASD continuum. Rates of diagnoses within the FASD continuum are then estimated from these in-school data via three different methods. The range of rates of FAS produced by these methods is between 4.0 to 12.0 per 1,000; Partial FAS ranges from 18.1 to 46.3 per 1,000; and an FASD was found in 2.3% to 6.3% of the children.

**Conclusions:**

These rates are substantially higher than previous estimates of FAS and overall FASD for the general populations of Western Europe and the U. S., and raise questions as to the total impact of FASD on mental deficit in mainstream populations of Western Europe and the United States where the majority are middle class and are not believed to be characterized by heavy episodic drinking.

## 1. Introduction

Moderate daily or frequent drinking is believed to be the predominant pattern of alcohol consumption among a majority of females in Italy. While individual cases of children with fetal alcohol syndrome (FAS) in Italy exist in the published literature [1–5], studies in Italy have frequently shown no relationship between maternal alcohol consumption and reduced birth weight or pregnancy loss [6–9]. But at least one article from Italy has linked prenatal alcohol use and smoking with low birth weight [10]. Another reported that over one-third of women delivering in Italian hospitals were daily drinkers, and that drinking continued after recognition of pregnancy [11]. Overall, maternal drinking in this later study [11] was not associated with lower birth weight. But birth weight was affected by “*abusive*” drinking, and alcohol abuse and binge drinking were cited as rare among women. Primatesta *et al.* [9] also reported low rates of pre-pregnancy binge drinking (1.4%) among women in Milan; however, their data indicate that 9% of women reported risky to very risky average weekly alcohol consumption, with 29% drinking daily during pregnancy, rates substantially higher than those reported in the United States (U.S.).

The majority of all studies of the prevalence of fetal alcohol spectrum disorders (FASD) are from the U.S., and most have utilized clinic [12,13] or record-based systems [14–16] without active recruitment. Such methods under-report prevalence and describe only the most severe cases [17,18]. Without active case ascertainment, many children with FAS and FASD are neither detected [16,19–21] nor referred for diagnosis [20,22–25]. Therefore, many cases of FASD, from FAS to alcohol-related neurodevelopmental disorder (ARND), have not been well-defined in the literature because of this selectivity in the referral process and the lack of active outreach [18].

In population-based, active case ascertainment studies, FASD cases are sought through structured outreach in a defined population [20]. All published, active case ascertainment studies in the U.S. except one [21], were carried out in predominantly minority and low socioeconomic status (SES) communities all of these utilized an active outreach system to refer children to specialty clinics [26–31]. Three prevalence studies utilizing in school studies of mostly low-SES populations have been reported from South Africa [32–37], studies which have been seminal in defining the value and techniques for in-school research methodology for FASD. This paper utilized in-school methods pioneered in South Africa.

A first wave of research in this area of Italy [38–40], revealed many alcohol-linked disabilities and a FAS prevalence of 3.7 to 7.4 per 1,000. Overall FASD was estimated as 35 per 1,000 (3.5%) [38], much higher than existing estimates for mainstream populations in a western country. As originally planned and funded, two samples (waves) of data were collected in the same region in order to sample more completely for FASD in this population. Complete data from both waves of this study in Italy are presented here to more fully address these questions: are FAS and other forms of FASD common in Italy; what are the characteristics of Italian children with FAS, or other diagnoses within FASD; and by implication, is the prevalence of FASD higher in the western world than estimated previously?

## 2. Methods

### 2.1. Institute of Medicine (IOM) Diagnostic Categories of FASD

The diagnostic components and criteria for FASD of the U.S. Institute of Medicine [20], recently revised slightly by the authors of this study [41], were used for classification of study children: (1) facial and other dysmorphology, (2) diminished physical growth, (3) intellectual, developmental, social, and neuropsychological assessments, and (4) maternal alcohol consumption, physical, and social risk factors. Also, known anomalies of genetic and other teratogenic origins are ruled out by clinicians (dysmorphologics/medical geneticists) before an FASD diagnosis is made. Data for each of the above components were collected and analyzed independently within each of the above domains. A formal, data-driven case conference was held for final diagnosis where the professionals representing each domain present the results obtained for each child. Thus, the diagnosis is made by the multidisciplinary team.

The diagnoses in the IOM system are: FAS, Partial FAS, alcohol-related neurodevelopmental disorder (ARND), alcohol-related birth defects (ARBD), or not FAS [20,41]. For FAS a child must have: (1) a characteristic pattern of minor facial anomalies including at least 2 or more of the key facial features of FAS (palpebral fissures ≤ 10th centile, thin vermilion border, or smooth philtrum), (2) evidence of prenatal and/or postnatal growth retardation (height or weight ≤ 10th centile), (3) evidence of deficient brain growth (structural brain anomalies or occiptofrontal head circumference (OFC) ≤ 10th centile), and if possible, (4) confirmation of maternal alcohol consumption from the mother or a knowledgeable collateral source [41].

For Partial FAS (PFAS), a child must have: (1) evidence of a characteristic pattern of facial anomalies including two or more of the three key features of FAS (above), (2) one or more other characteristics such as prenatal or postnatal growth retardation (≤10th centile in height or weight), hypoplastic midface, occilar defects, abnormalities of the fingers, or other physical defects linked to prenatal alcohol exposure in humans, (3) small OFC (≤10th centile), and/or evidence of a complex pattern of behavioral or cognitive abnormalities inconsistent with developmental level and unexplainable by genetic composition, family background, or environment alone; and if possible, (4) direct or collateral confirmation of maternal alcohol consumption [41].

For a diagnosis of ARND a child must have documentation of significant prenatal alcohol exposure, display neurological or structural brain abnormalities (e.g., microcephaly), or manifest evidence of a complex and characteristic pattern of behavioral or cognitive abnormalities inconsistent with developmental level as measured by test batteries such as those in Table 2 and not explained by genetic predisposition, family background, or environment alone [41].

For ARBD, a child must have confirmed prenatal alcohol exposure, evidence of the characteristic pattern of facial anomalies, including two or more of the following: short palprebral fissures, thin vermillion border, and/or smooth philtrum, as well as either major malformations of a pattern or minor malformations, but generally rather normal neurobehavioral performance [41].

Diagnosis of FAS or PFAS without a confirmed history of alcohol exposure must be viewed as tentative, but original IOM criteria allow for an FAS diagnosis without direct (maternal) reports of exposure [20], and our revised criteria [41], permit it in a diagnosis of PFAS if all other signs point consistently to a regular pattern of drinking at other times (e.g., prior to being told of pregnancy, and 3 months prior to pregnancy). Collateral reports are valuable sources of information which were utilized, but could not be obtained for all mothers not interviewed. Many women in well-educated, developed populations do not admit to drinking during pregnancy [42].

### 2.2. Overall Study Design and Sampling

First grade students were studied from two health districts of the Lazio region outside of Rome, an area of small towns, some suburban, and other relatively self-sufficient, rural areas. These health districts are considered relatively representative of central Italy.

The study is a cross-sectional, observational, case-control design with retrospective collection of maternal risk information. Twenty-five schools were randomly selected in wave I (2005–2006), from the 68 elementary schools in two health districts, and 25 were selected in wave II (2006–2007). Random sampling with replacement resulted in double selection of seven schools who participated in both years; thus, 43 different schools participated. Italian team members approached school administrators to explain the study, gain entry, and contact parents/guardians of all first grade children via normal school communication channels. Total enrollment in the first grade of selected schools was 1,087 and 902 in waves I and II. Positive consent forms were returned by 49% of the parents with only one mail out. Subsequent distributions of consent forms will generally yield a higher percentage of participation, but this study was severely limited by time and monetary resources, so one mailout was all that was possible.

The total sample was 976 children; 11 of the initially consented children were withdrawn by their parents at some later point in the study or they moved from the study schools. All procedures were approved by the Ethics Committee of the Italian health district (ASL RMG) and the University of New Mexico Health Sciences IRB (approval #03 089).

The randomly-selected control children were chosen by random number tables from all consented children to provide a representative population of normal, not-FASD children. True to the population-based nature of this study they represent children of all sizes and attributes, including both those children who are exposed and unexposed to alcohol during pregnancy. Nevertheless, no control children have an FASD or any other major defect or anomaly. In any given population, especially in certain European populations where some drinking among women is practiced almost universally, there are many completely normal children who are exposed to some alcohol in the prenatal period, yet they do not suffer any detectable anomalies or deficits from the exposure. So the controls in this and our other population-based studies are representative of the normally-functioning children in these schools.

Because the first tier screening in this in-school study primarily sought children who were small or had small OFC, we were most likely to detect children with a severe form of FASD, e.g., FAS and PFAS. That was our intent. Other than the children who were specially referred by teachers as having learning or behavioral problems, we undoubtedly missed cases of ARND. Monetary and time limitations dictated that we limit our first tier screening to a small number (≤10th centile on OFC and/or height and weight) in order to locate the most affected. Teacher referrals were encouraged by the research team and were made for children whom the teachers believed were struggling with their lessons, were behaviorally disordered, or showed some other signs of having features of FASD.

### 2.3. The Two-Tiered System of Entry and Diagnosis Employed in the Study

**Figure 1.**
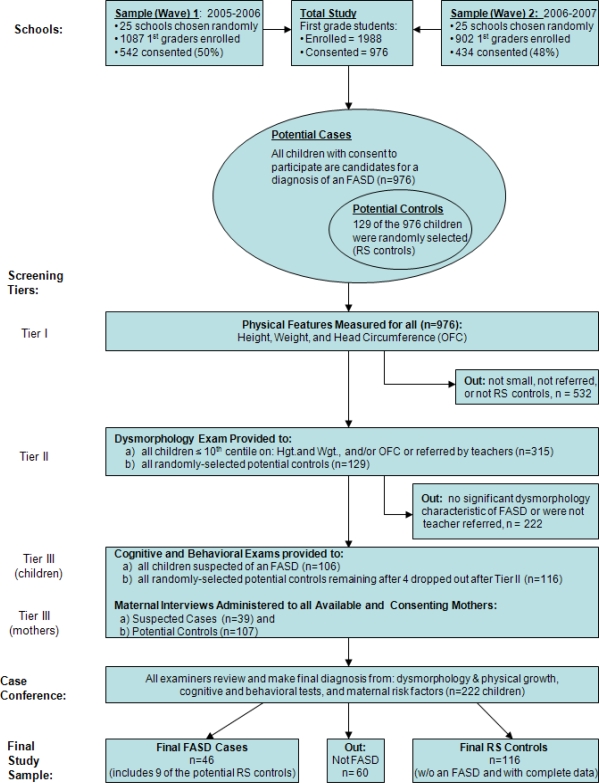
Methodology of the Lazio Region (Italy) FASD study with Sampling Procedures and Numbers.

Data on growth, development, and dysmorphology were collected by a two-tiered method, and US National Center for Health Statistics growth charts were used to determine the centiles for each child. In Tier I, consented children were screened for height, weight, and head circumference (OFC) in their schools. If a child’s measurements were ≤10th centile on OFC and/or height and weight, he/she was referred for Tier II screening, which began with the dysmorphology exam in each school by dysmorphologists and research staff. Also, teachers were asked for and provided referrals of children with learning problems who were included in Tier II if consented. Six dysmorphologists worked with checklists to assess growth and morphology [41]; each child was examined by two physicians independently, scribes recorded data, and all were blinded from information from all other sources. Findings of the two examiners were later compared for each child during daily debriefing sessions where a preliminary diagnosis was assigned: probable FAS, deferred-suspected FASD, or not FAS. Children with a preliminary diagnosis of any possible FASD (n = 118) were advanced to neurobehavioral testing (See Figure 1).

### 2.4. Developmental (IQ, Cognitive and Behavioral) Testing for FASD Suspects and Controls

Randomly-selected control children (n = 116) from the same classrooms were provided identical physical exams and neurobehavioral testing as provided to the suspects by local professionals. Neurobehavioral testing was carried out by licensed Italian psychologists and the testing included: Rustioni Qualitative Test [43], Raven’s Colored Progressive Matrices [44], the Italian translation of the Wechsler Intelligence Scale for Children–Revised (WISC-R) [45] the Personal Behavior Checklist (PBCL) [46], the Pelham Disruptive Behaviors Disorder (DBD) Scale (filled out by both parents and teachers) [47], and the Questionario Osservativo per I’Identificazione Precoce delle Difficultà di Aprendimento (IPDA) [48].

The Rustioni Qualitative Test is a measure of Italian language linguistic understanding. It was developed and normed on the Italian population. The IPDA is an Italian normed test designed to identify difficulties in learning by measuring academic achievement. The Raven CPM is a standardized test that assesses nonverbal reasoning ability. The PBCL is a short, easy to administer scale that purports to measure the behavioral characteristics of FAS, regardless of age, race, sex or IQ. The DBD Rating scale provided measures of inattention and hyperactivity/impulsivity.

### 2.5. Maternal Interviews for Mothers of FASD Suspects and Controls

Thirty-nine of 46 mothers of the children with final diagnoses of an FASD (85%) were interviewed on maternal risk factors exploring their lives before, during, and after the index pregnancy including: childbearing, drinking, drug use, marital status, SES, demographics, religiosity, and nutrition. The 107 (92%) consenting mothers of the control children became the maternal comparison group.

### 2.6. Statistical Analysis

Data were processed via EPI Info software [49] and SPSS. In Tables 1 and 2 one-way Analysis of Variance is used to compare children with severe FASD and controls. Dunnett’s C tests were used for *post-hoc* analyses. Table 3 employs t-tests and chi-square to compare mothers of FASD subjects combined with control mothers. Zero order correlations (Pearson r) of selected variables are reported in the text after Table 3. In Table 4, FASD prevalence estimates are presented using two denominators, high (sample as the denominator) and low (total enrolled children), as specified in the table footnotes. Finally, alternative estimates of FASD are presented in the text using the conversion rates of controls to an FASD diagnosis.

## 3. Results

### 3.1. Demographic and Child Physical Traits

Table 1 compares demographic and physical traits of study children. Because the focus of this study was on the most severe forms of FASD, one child with ARND and one with ARBD were omitted in Tables 1 and 2. But they are included in Table 4 for the total prevalence of FASD. There is no difference in the groups by age and sex, reinforcing the strength of in-school studies utilizing randomly selected controls for these variables. Also, the values for the control group are similar to those collected for the whole sample, indicating that random methods produced a representative sample. Specifically, children with FAS have the most significantly deficient measures of height, weight, BMI, head circumference, short intercanthal distance, hypoplastic midface, strabismus, narrow vermillion border, maxillary arc, mandibular arc, and heart murmur, while the controls have the best measures as expected from both the study design and criteria for FASD. The spectrum effect is evident, with FAS children having the most anomalies and worst growth and development, the features of the children with PFAS are less severe, and controls are relatively unaffected. But slight exceptions to the spectrum effect exist with some significant variables: palpebral fissure length (FAS and PFAS are equally small), philtral length (PFAS and FAS equally long), ptosis (PFAS have the only feature here) and smooth philtrum (FAS and PFAS have equally high percentages).

Nevertheless, with each of these later variables, F-values of difference were significant between the three groups and measures for the FAS, and as predicted by the literature PFAS children are worse than controls [20,41]. Total dysmorphology scores (overall severity of deformities and lack of physical development) clearly form the expected spectrum (FAS = 15.8, PFAS = 11.2, and 3.6 for controls).

### 3.2. Neurodevelopmental Traits of Children

Scores on neurodevelopmental tests (Table 2) indicate average scores, non-verbal I.Q. (Raven) and verbal, comprehension (Rustioni), and academic understanding (IPDA) are significantly lower for FAS children than PFAS and controls. While there are no *post-hoc* significant differences between FAS and PFAS IQ on these measures, a significant difference exists between the PFAS and controls. The WISC-R non-verbal IQ scale scores form a significant spectrum with FAS children scoring the lowest (85.5), PFAS next (95.4), and controls the highest (113.7). The Pelham Disruptive Behaviors Disorder (DBD) data in Table 2 are the parent ratings from these children. Parents rated the FASD children as less attentive than controls, and *post-hoc* analyses show the PFAS children to be significantly less attentive than controls. Overall, hyperactivity was greatest among the PFAS group and lowest among the FAS group, the latter being an unusual finding in the literature. *Post-hoc* analyses of hyperactivity were not significantly different between groups and are not presented in Table 2. Italian parents seem less likely than American parents to rate children as hyperactive. PFAS children also registered the greatest number of behavioral problems on the PBCL, and the FAS and control children have identical scores. Finally, classroom academic achievement (IDPA) is lowest for FAS children, with PFAS children intermediate; controls are doing the best.

### 3.3. Maternal Traits

Not all mothers of FASD children reported drinking during the index pregnancy (Table 2), but mothers of the FASD children reported significantly more drinks per week and per day at interview (“current drinking”). Notably, mothers of FAS children reported the greatest quantities of current drinking, as current drinking variables preceded questions of prenatal drinking. Like many other studies of FASD, mothers of the FAS and PFAS children were older, but the difference was not significant in this relatively small sample.

Table 3 indicates that virtually all mothers are current drinkers (93 to 97%). Mothers of children with FASD report heavy current drinking and drinking during the 2^nd^ and 3^rd^ trimesters of the index pregnancy. Lack of candor among a substantial number of the mothers of affected children was reported by the interviewers. No smoking variables were significantly different between groups. Some other significant variables include: mothers of FASD children were lower in educational attainment, higher in religiosity, and had lower status employment. No other differences were significant, but most were in a direction predicted in the literature.

### 3.4. Correlation Analyses

There were some important significant zero-order correlations produced in this study which link mother’s current drinking and their children’s inattention scores on the DBD scale (*r* = 0.19; *F* = 5.03; 2/143; *p* < 0.05) and dysmorphology score (*r* = 0.19; *F* = 5.67; 2/142; *p* < 0.05). Similar significant correlations linked maternal weight and the child’s non-verbal I.Q.; the higher the mother’s weight the higher the child’s I.Q. (*r* = 0.16; *F* = 3.92; 2/143; *p* < 0.05). For the children: head circumference (*r* = 0.27; *F* = 12.72; 2/142; *p* < 0.001), smooth philtrum (*r* = −0.28; *F* = 18.56; 2/142; *p* < 0.001), narrow vermilion (*r* = −0.21; *F* = 7.09; 2/143; *p* < 0.01), ptosis (*r* = −0.16; *F* = 3.90; 2/141; *p* < 0.05) and total dysmorphology score (*r* = −0.23; *F* = 9.14; 2/143; *p* < 0.01) are all significantly correlated with the child’s non-verbal I.Q.

### 3.5. Final Diagnosis Summary

#### FAS

In this Italian sample, 50% of the children diagnosed with FAS in the final case conference had all three “cardinal” or key facial features of FAS (short PFL, smooth philtrum, and narrow vermilion border). The other 50% had two of these facial features, often along with other facial dysmorphia (e.g., wide intercanthal distance). Furthermore, all children (100%) diagnosed with FAS met criteria for growth retardation and OFC at <10th centile. For the drinking data, 100% of the mothers of FAS children were determined to be drinkers through collateral reports or self-report. All of the mothers of FAS children who reported drinking (63%) in their lifetime reported drinking at 3 months prior to pregnancy, and of those who reported drinking during the index pregnancy, (after they found out they were pregnant), 50% reported drinking in all three trimesters and 25% in two trimesters and 25% in one. In the final diagnoses the five children born to mothers who reported drinking in the index pregnancy are reported as “confirmed alcohol exposure,” and the other three are reported in Table 4 as “without direct confirmation of alcohol use during pregnancy.”

#### PFAS

For this sample 33% of the children with PFAS had all three key facial features and 67% had two. Most PFAS children had other features of facial dysmorphia, e.g., hypoplastic midface, ptosis, or palpebral fissures (PFL) that were small (low %) compared to the innercanthal distance (ICD). Their overall dysmorphology scores were also high (11.2 ± SD of 4.0) for PFAS cases indicating other minor anomalies associated with prenatal alcohol exposure and FASD (Table 1). All of the PFAS cases diagnosed had scores on the intelligence, cognitive, and behavioral measures which were significantly deficient or problematic (Table 2). All but seven of the mothers of PFAS children were interviewed and of those who reported drinking at any time in their lives, 87% reported drinking during the 3 months prior to the index pregnancy; 58% of all mothers specifically reported drinking after their pregnancy was confirmed. Seventy-five percent of the mothers who reported drinking by trimester confirmed drinking in all three trimesters, the other 25% in only 1 or 2 trimesters. If a mother of a child with PFAS did not directly report drinking during the index pregnancy, then the case is listed in Table 4 as “diagnosis without direct confirmation of alcohol use during pregnancy,” even though in some cases collateral reports may have indicated alcohol use during pregnancy.

The one child diagnosed with ARND had minimal dysmorphology consistent with FAS or PFAS, yet his mother reported heavy drinking and his pattern of cognitive and behavioral traits was significantly deficient from normal, and consistent in pattern or profile with the children with FAS or PFAS. The child diagnosed with ARBD has substantial dysmorphology, but relatively average I.Q. and behavioral performance.

### 3.6. Prevalence Findings

In Table 4 the prevalence findings and the estimates for the range of FASD diagnoses are presented utilizing several techniques. First, the sample rates are calculated using the denominator of total consented children (n = 976). These are presented in column “a”. The rate of FAS is 8.2 per 1,000 children, the rate PFAS is four times higher than FAS at 36.9 per 1.000 and the overall rate of FASD is 47.1 per 1,000 using these sample data. Furthermore, in column “b”, 95% confidence intervals (CI) were calculated from the two cohort samples yielding a range in the sample rates of: 6.5 to 10.1 per 1,000 for FAS, 32.7 to 40.6 per 1,000 for PFAS, and 33.4 to 62.6 per 1,000 for total FASD.

The second method of estimation is found in column “c” of Table 4, where calculations are based on a denominator of the total children enrolled in the first grade classrooms (n = 1,988). This represents a conservative prevalence estimate, and this technique is warranted given the fact that over sampling of children was undertaken in this study by including all children in the “at risk” categories of ≤10th centile on height, weight, and head circumference and those referred by teachers as suspected of having behavioral or learning problems. The rates utilizing this technique are: 4.0 per 1,000 for FAS, 18.1 per 1,000 for PFAS, and 23.1 per 1,000 for all FASD cases found. In column “d” then, a wider range of estimates is found, where the higher numbers are the average sample rates and the lower rate is that estimated from all enrolled children: 4.0 to 8.2 per 1,000 FAS, 18.1 to 36.9 per 1,000 PFAS, and 23.1 to 47.1 per 1,000 for total FASD.

Third, a single rate estimate can be produced by projecting the positive diagnosis rate of FASD found among the consented randomly-selected children to the number of children in the non-consented portion of the population. If the prevalence of various FASD diagnoses occur at the same rate in the non-consenting population as in those that were randomly-selected from the consented population, then we have another reasonable and accurate way of estimating the prevalence of FASD and individual diagnoses within. Nine consented children, selected originally as random controls, converted to a diagnosis within the spectrum of FASD: two converted to FAS and seven to PFAS (see column “e” in Table 4). Therefore, 71 per 1,000 or 7.1% of the original 126 (9/126) consented children picked initially as candidates for normal controls converted to an FASD diagnosis. When projected to the 1,012 children not provided consent, 72 additional cases are estimated. When added to the diagnosed cases in the consented population the yield is an FASD rate of 59.4 per 1,000 (or 5.9%). Using the same projection method applied to FAS only, the single rate of FAS may be 12.0 per 1,000 or 1.2% (see column “f” in Table 4), and the single rate of PFAS is 46.3 per 1,000 or 4.3%. The widest range of estimated rates are summarized in column g.

## 4. Discussion

### 4.1. Prevalence Findings

In this study we have used well-established, active case ascertainment, in-school study methods and techniques employed by a highly experienced team of classical and epidemiologic researchers to gather evidence on the prevalence of specific diagnoses within the continuum of FASD. Forty-six first grade children in the central study schools were found to have a diagnosis within the FASD continuum for their physical, developmental, and prenatal histories were found to be consistent with cases found elsewhere in the world. Because of the population-based sampling methods employed, we are able to utilize these empirical methods to estimate the prevalence of FASD within these communities. Using three calculation methods, the rates of FAS and total FASD were calculated. First, the sample rate of FAS was the highest at 8.2 per 1,000 and a 95% CI range of rates for FASD of 33.4 to 62.6 per 1,000. Second, the rate for all enrolled children produced by the over-sampling (of small children) method employed was lower at 4.0 per 1,000 for FAS and a range of 23.1 to 47.1 for FASD. Finally, the random sample of the consented children produced a high rate of FAS at 12.0 per 1,000 and a single, rate of FASD at 59.4 per 1,000, but a rate which is high within the sample 95% confidence intervals. Therefore combining the range of rates from the three estimates, the rate of FAS in this central Italian population is between 4.0 to 12.0 per 1,000 and the rate of FASD is between 23.1 and 62.6 per 1,000 or 2.3% to 6.3%.

### 4.2. Implications

The high rates of FASD, particularly FAS and PFAS, presented here are provocative. They are substantively higher than the often quoted rates of 0.5 to 3.0 per 1.000 for FAS and 1% for FASD [18]. We note here that none of our experience with the methods used, or the presentation of clinical traits of children in Italy, was atypical of our experience in studies implemented in large samples in South Africa [32,35,37], with referral clinics in the U.S. [26–28,31], or similar in-school pilot studies underway in a Western U.S. city [18]. Recent advances in diagnostic methods and skills made in recent studies have clarified the criteria for diagnosing and recognizing FASD [41]. We believe that the rates of FAS and PFAS presented here are accurate, and indicate that the prevalence of FASD is higher in Italy than previously believed. Clinically, our team found the presentation of the FASD cases to be similar in their characteristics to those of other populations we have worked in and studied.

Because the risk factors for FASD are more severe in study populations in South Africa, where most in-school studies have occurred, and the occurrence of FAS and PFAS is substantially more frequent there (46 to 89 per 1,000) [32,34,36], direct comparisons of these FASD rates and characteristics in Italy to South Africa are less useful than would be a comparison to other highly-developed populations. But there are few reported active case ascertainment studies in Europe and the U.S. In the one pilot study carried out in schools in the U.S., only FAS was assessed. The rate reported was 3.1 per 1,000 [21], certainly close to the FAS prevalence reported in this study from the total enrollment and higher than rates reported by other methods [18]. Interestingly only one of the seven children with FAS reported in that U.S. study [21] had been diagnosed previously. As far as we know, none of the FAS children diagnosed in this Italian study had been previously diagnosed. Our literature review yielded only 24 cases of FAS reported in the Italian literature prior to our first wave of research in Italy in 2004 [38]. Additionally, an intensive, prospective, clinic-based study by Sokol *et al.* [50] in Cleveland also reported 3.1 FAS cases per 1,000. But overall, much lower rates have been reported by surveillance and clinic-based studies because FASD is difficult to diagnose (and is often missed) in newborns and infants [18,51].

This study utilized population-based screening to examine the prevalence and characteristics of FASD in a general population of Italy where the majority of the subjects were middle class and there was not a large disparity found in maternal social class or maternal education between the two groups, cases and controls. While one can argue that not having measures of maternal I.Q. might bias the neurobehavioral findings, previous explorations of such bias have not found a major influence on the verbal or non-verbal I.Q. or behavior problems of the children in this study [39,40]. Valuable insights into the risk for FASD in a Western European population were gained from this study. As might be expected in a population where heavy episodic (binge) drinking is not common, there were 4.5 cases of PFAS for each case of FAS. Moderate daily drinking may produce fewer severely dysmorphic cases within FASD, but this study clearly demonstrates that children of more moderate daily drinkers do suffer from increased dysmorphia and disabilities compatible with FASD.

### 4.3. Limitations

Certainly there are limitations to this study. First, reporting of prenatal drinking is imperfect in this and in maternal populations with a relatively high degree of education [42]. The maternal interviewers for this study stated that the low rate of drinking reported during these pregnancies represented substantial underreporting in their opinions. Therefore some cases of ARND may well have been missed. Nevertheless, current drinking measures and retrospective, pre-pregnancy drinking reports provide additional useful information and checks for validity. Also, revised IOM criteria allow the diagnosis of FAS and PFAS without direct maternal reports of drinking when the specific dysmorphic traits found in a particular child are those that have been clearly linked to FASD in other species and in other human populations [32–37,51–54]. And collateral reports have proven valuable. But our ability to diagnosis ARND and ARBD are severely hampered by lack of reporting. Second, the participation rate in this study was not as high as desired (49%). Limitations of time and money only allowed for one permission slip per child to be sent home in each wave of research. Researchers attempted to account for this problem by recruitment and active follow-up of referred children believed by teachers to have learning and behavioral problems, thus ensuring greater capture of probable cases. Furthermore, the range of rates reported here attempts to correct for incomplete recruitment by assuming that the rate of FASD in the non-consented children was at least as high as for participants and a projection of the population of FASD found by random case selection to the non-consented children. Third, some I.Q. and behavioral tests that were desired for comparing Italian children to English-speaking populations were not translated or standardized in Italy, and, therefore, could not be used. Also, some American behavioral measures, such as the PBCL, were apparently utilized differently by Italian parents, for problem scores reported were substantially lower for all Italian children than those normally reported by Americans. Fourth and finally, because this study began first with and was primarily based on screening and exams for dysmorphic features and poor physical growth, the cases of ARND are severely undercounted. Using a different methodology which would begin first with behavioral and neurocognitive testing would likely yield substantially more cases of ARND. Also, using totally random methods might produce more children with ARND and minimal dysmorphia.

## 5. Conclusions

The rates of FAS and PFAS among the children in this region of Italy are high compared to previous estimates for the Western world. Since the currently accepted rates of FAS in the U.S. are 0.5 to 3.0 per 1,000 [20,26], and total FASD is estimated at 1% [13], the rates of FAS and FASD in these Italian communities are significantly higher than previously estimated for a developed country [18,22–24]. No matter which of the rates produced here by various methods are used, the currently accepted rates for the U.S. and Europe seem to be unrealistically low.

Insight has also been gained into the FASD implications for the Italian drinking style. Regular drinking during meals in a well-nourished and well-educated maternal population, (as opposed to a poorly nourished, low SES, binge drinking population), can produce a number of children with FASD. The drinking style of some women in this study obviously exceeds quantities and frequencies that are safe in this population. Children with an FASD present substantial challenges to any and all parents, schools, clinicians, and societies; thus, there is a need to identify such children early so that their development and normal adaptive skills can be maximized and FASD prevention initiated for individual mothers, families, and larger groups of aggregates. This message may and should resonate with other Western European populations and also for the U.S. population, for we feel that this study has carefully examined a population with social and economic characteristics similar to thousands of others in the western world.

## Figures and Tables

**Table 1.** Demographic and growth parameters for all study children, children with a final diagnosis of FAS, partial FAS, and randomly selected controls: Lazio Region, Italy.

**Variable**	**Children In Study (n = 970)**	Children With FAS (n = 8)****	**Children With PFAS (n = 36)**	**Control Children (n = 116)**	***P***
Sex (%)					
Males	50.6	37.5	52.8	52.6	
Females	49.4	62.5	47.2	47.4	NS (0.706)^a^

Age (months)					
Mean (SD)	79.5 (4.2)	80.9 (2.9)	79.4 (4.3)	79.5 (3.5)	NS (0.577)^b^

Height (cm)*					
Mean (SD)	121.5 (5.5)	113.6 (3.6)	118.0 (5.0)	121.5 (4.9)**	<0.001^b^

Weight (kg)*					
Mean (SD)	25.1 (5.2)	18.8 (3.1)	22.2 (3.7)	25.1 (4.2)**	<0.001^b^

Children’s BMI*					
Mean (SD)	16.8 (2.8)	14.6 (2.9)	15.9 (1.76)	16.9 (2.3)	0.002^b^

BMI Percentile*					
Mean (SD)	60.9 (31.2)	20.6 (32.6)	51.4 (29.0)	65.6 (29.4)	<0.001^b^

Occipital Circumference (OFC) (cm)*					
Mean (SD)	51.9 (1.5)	49.1 (1.0)	50.6 (1.7)	52.0 (1.3)**	<0.001^b^

Palpebral Fissure Length (PFL) (cm)					
Mean (SD)		2.4 (0.1)	2.4 (0.1)	2.5 (0.1)	<0.001^b^

Short Innercanthal Distance (ICD) (≤25%)		25.0	19.4	6.0	0.022

Percent PFL is of ICD					
Mean(SD)		84.0 (10.4)	87.9 (9.9)	89.4 (7.2)	NS (0.139)^b^

Philtrum Length (cm)					
Mean		1.5 (0.2)	1.5 (0.2)	1.4 (0.2)	0.035

Hypoplastic Midface (%)		62.5	30.6	12.9	0.001 ^a^

Maxillary Arc (cm)					
Mean		23.3 (0.5)	24.4 (1.1)	24.9 (0.9)	<0.001

Mandibular Arc (cm)					
Mean (SD)		23.9 (0.5)	24.9 (1.2)	25.6 (1.0)	<0.001

Strabismus (%)		12.5	5.6	1.7	0.0034^a^

Ptosis (%)		0.0	8.3	0.0	0.005^a^

Smooth Philtrum (%)		87.5	91.7	13.8	<0.001^a^

Narrow Vermillion Border (%)		100.0	94.4	21.6	<0.001^a^

Heart Murmur (%)		12.5	0.0	1.7	NS (0.060)^a^

Clinodactyly (%)		50.0	55.6	36.2	NS (0.105)^a^

Camptodactyly (%)		0.0	13.9	4.3	NS (0.088)^a^

Dysmorphology Score***					
Mean (SD)		15.8 (1.9)	11.2 (4.0)	3.6 (2.9)	<0.001^b^

NS = Not Significant.

a. χ^2^ test of data comparing children with FAS, PFAS, and controls; a Fisher’s exact test when there are cells with an expected value of less than five.

b. ANOVA of data comparing children with FAS, PFAS, and controls.

* Measurements are actual values at the time of screening and exams. Percentiles were calculated via standardized NCHS growth charts for age and sex and used (1) when considering inclusion of children in the study, (2) for comparison, and (3) when diagnosis was made.

** Measurements at time of Tier I screen, therefore they are directly comparable to all other groups.

*** The dysmorphology score is a weighted measure of dysmorphic features. It is not utilized in diagnostic assessment, but provides a quantitative measure of dysmorphic features for comparison purposes [41].

**** There was one set of twins among the FASD cases.

**Table 2.** Demographic and behavioral indicators of controls and children with a FASD diagnosis and comparisons of maternal age and drinking measures across groups: Lazio Region, Italy.

	Final Diagnosis FASD					
			
	FAS Mean Score (SD)	Partial FAS Mean Score (SD)	Controls Mean Score (SD)	Test Statistic ***	df	probability
**Child Variables**	(*n* = 8)	(*n* = 36)	(*n* = 113)			

Total dysmorphology score	15.75^bc^ (1.90)	11.22^ac^ (3.95)	3.60^ab^ (2.90)	*F* = 122.76	2/160	<0.001
Raven percentile	50.62 (28.71)	55.55^c^ (22.28)	70.97^b^ (21.20)	*F* = 8.99	2/157	<0.001
Rustioni errors	8.00 (3.91)	7.76^c^ (1.88)	5.27^b^ (2.51)	*F* = 8.37	2/85	<0.001
Rustioni qualitative	3.13 (2.03)	3.56^c^ (2.09)	4.78^b^ (1.74)	*F* = 8.00	2/156	<0.001
Inattention DBD	5.12 (7.01)	7.28^c^ (8.10)	2.21^b^ (3.72)	*F* = 12.97	2/154	<0.001
Hyperactivity DBD****	1.75 (2.71)	4.86 (6.71)	2.19 (4.26)	*F* = 4.23	2/156	0.016
WISC-R Non-verbal IQ****	85.50 (22.21)	95.42 (15.45)	113.69 (17.48)	*F* = 11.03	2/71	<0.001
PBCL****	4.80 (5.76)	7.37 (6.84)	4.80 (5.76)	*F* = 6.20	2/136	0.003
IDPA total score	15.50 (4.98)	17.03 (4.64)	21.11 (5.78)	*F* = 9.68	2/151	0.001

**Maternal Variables**	(*n* = 8)	(*n* = 34)	(*n* = 112)			

Maternal age during index pregnancy						
Mean (SD)	31.50 (6.00)	30.48 (5.20)	29.25 (5.38)	*F* = 1.11	2/145	NS(0.331)
Report drinking during pregnancy (%)	50.0	54.8	40.0	χ^2^ = 2.28	2/144	NS(0.320)
Mean number of drinks current week** (SD)	10.37 (18.92)	1.78 (4.02)	1.52 (2.80)	*F* = 10.94	2/145	<0.001
Mean drinks per current drinking day** (SD)	1.56 (2.69)	.63 (.53)	.61 (.52)	*F* = 5.39	2/145	0.006

* = all scores standardized for age of child at time of testing.

** = Among those who reported drinking during pregnancy; includes current non-drinkers.

*** = Univariate Analysis of Variance (ANOVA) or chi-square.

**** = Ratings performed by parents. *Post-hoc* analysis, significantly different from:

FAS = a,

PFAS = b,

Controls = c: Dunnett’s C adjustment, the mean difference is significant at the 0.05 level. NS = not statistically significant.

**Table 3.** Demographic, socioeconomic, and maternity variables and substance use measures by mothers of the children with FASD and randomly selected controls: Lazio Region, Italy.

**Variable**	**Mothers of children with FASD (n = 39)**	**Control mothers (n = 107)**	**Test statistic *P***	**OR (95% CI)^d^**
**Demographic and Socioeconomic Variables**				

Mean Age (yrs) on day of interview (SD)	37.2 (5.3)	36.1 (5.4)	NS (0.260)^b^	
Educational attainment (%)				
Elementary	7.7	1.9		
Junior high	46.2	27.1		
Senior high	23.1	51.4		
Beyond Senior High	5.1	1.9		
College Degree	17.9	17.8	0.015 ^a,e^	
Religiosity Index - Mean (SD)	4.8 (2.1)	3.9 (2.2)	0.025^b^	
Among those employed, actual job (%)				
Manual worker	50.0	23.5		
Office worker	44.4	54.4		
Manager in an office	0.0	1.5		
Manager	5.6	19.1		
Other	0.0	1.5	NS (0.212)^a,e^	

**Substance Use Variables**	(n = 30)	(n = 78)		

Current drinker^1^ (of ever drinkers) - %	93.3	97.4	NS (0.311)	0.37 (0.03–3.95)
Mean number of drinks last month^2^	20.3 (46.4)	8.7 (12.0)	0.045^b^	
(current drinkers) (SD)				
Among ever drinkers, drinking during:				
1^st^ trimester of pregnancy with index child - %	53.3	36.7	NS (0.115)^a^	1.97 (0.77–5.08)
2^nd^ trimester of pregnancy with index child - %	60.0	34.2	0.014^a^	2.89 (1.11–7.60)
3^rd^ trimester of pregnancy with index child - %	56.7	34.2	0.033^a^	2.52 (0.97–6.56)
Current smoker (of those who ever smoked) - %	34.8	57.7	NS (0.067)^a^	0.39 (0.12–1.22)
Percent smoked 3 months before index pregnancy (among ever smokers)	73.9	71.2	NS (0.806)^a^	1.15 (0.33–4.07)
Percent who smoked during index pregnancy(ever smokers)	31.8	28.8	NS (0.798)^a^	1.15 (0.34–3.87)

1. Consumed alcohol in 12 months preceding interview.

2. Includes those who did not drink in past month. NS = not statistically significant.

a. *X*^2^ test.

b. *t*-test.

c. Difference of proportions test.

d. 95% confidence intervals calculated via the Cornfield technique.

e. Calculations of chi-square-based odds ratio not possible for this variable as it is not a 2 × 2 configuration.

**Table 4.** Cases diagnosed and estimated rates of FASD among first grade school children in the Lazio Region, Italy.

	**Diagnosis with direct confirmation of alcohol use during pregnancy**	**Diagnosis w/o direct confirmation of alcohol use during pregnancy**	**Total cases**	(a)* Sample rates*	(b)** 95% Conf. Interval*	(c)*** Rate for all enrolled children	(d) Range of rates for all enrolled children*	**(e) Cases in randomly selected group**	(f) **** Sample rate from random selection	(g) ***** Widest range of rates from various methods
FAS	5	3	8	8.2	6.5–10.1	4.0	4.0–8.2	2	12.0	4.0–12.0
PFAS	21	15	36	36.9	32.7–40.6	18.1	18.1–36.9	7	46.3	18.1–46.3
ARBD^1^	1	0	1	1.0	-	0.5	0.5–1.0	-	0.5	0.5–1.0
ARND^1^	1	0	1	1.0	-	0.5	0.5–1.0	-	0.5	0.5–1.0
Total	28	18	46	47.1	33.4–62.6	23.1	23.1–47.1	9	59.4	23.1–62.6

* Rate per 1,000 children based on the sample screened, denominator = 976.

** 95% confidence intervals were calculated via the two different, independent samples of different 1^st^ grade cohorts as discrete.

*** Rate per 1,000 children enrolled in first grade classrooms which assumes that oversampling of small children and teacher-referred children included a majority of “at risk” cases.

**** Rate per 1,000 children based on those selected at random for possible controls and after testing some children were found to have an FASD. Rate of children converting to an FASD diagnosis projected to the non-consented population then added to cases found in the consented population and divided by the total enrolled population.

***** Widest range of rates calculated from the various methods employed in Table 4.

1 ARBD and ARND cannot be diagnosed without direct confirmation of maternal alcohol use in the index pregnancy.
